# Allograft inflammatory factor 1 is a regulator of transcytosis in M cells

**DOI:** 10.1038/ncomms14509

**Published:** 2017-02-22

**Authors:** Sari Kishikawa, Shintaro Sato, Satoshi Kaneto, Shigeo Uchino, Shinichi Kohsaka, Seiji Nakamura, Hiroshi Kiyono

**Affiliations:** 1 Division of Mucosal Immunology, The Institute of Medical Science, The University of Tokyo, Tokyo 108-8639, Japan; 2 Section of Oral and Maxillofacial Oncology, Division of Maxillofacial Diagnostic and Surgical Sciences, Faculty of Dental Science, Kyushu University, Fukuoka 812-8582, Japan; 3 International Research and Development Center for Mucosal Vaccines, The Institute of Medical Science, The University of Tokyo, Tokyo 108-8639, Japan; 4 Core Research for Evolutional Science and Technology, Japan Science and Technology Agency, Tokyo 102-0076, Japan; 5 Mucosal Vaccine Project, BIKEN Innovative Vaccine Research Alliance Laboratories, Research Institute for Microbial Diseases, Osaka University, Osaka 565-0871, Japan; 6 Mucosal Vaccine Project, BIKEN Center for Innovative Vaccine Research and Development, The Research Foundation for Microbial Diseases of Osaka University, Osaka 565-0871, Japan; 7 Department of Neurochemistry, National Institute of Neuroscience, Tokyo 187-8502, Japan; 8 Department of Immunology, Graduate School of Medicine, Chiba University, Chiba 260-8670, Japan

## Abstract

M cells in follicle-associated epithelium (FAE) are specialized antigen-sampling cells that take up intestinal luminal antigens. Transcription factor Spi-B regulates M-cell maturation, but the molecules that promote transcytosis within M cells are not fully identified. Here we show that mouse allograft inflammatory factor 1 (Aif1) is expressed by M cells and contributes to M-cell transcytosis. FAE in *Aif1*^*−/−*^ mice has suppressed uptake of particles and commensal bacteria, compared with wild-type mice. Translocation of *Yersinia enterocolitica*, but not of *Salmonella enterica* serovar Typhimurium, leading to the generation of antigen-specific IgA antibodies, is also diminished in Aif1-deficient mice. Although β1 integrin, which acts as a receptor for *Y. enterocolitica* via invasin protein, is expressed on the apical surface membranes of M cells, its active form is rarely found in *Aif1*^*−/−*^ mice. These findings show that Aif1 is important for bacterial and particle transcytosis in M cells.

## Abstract (web summary)

M cells are intestinal epithelial cells that are specialized to transcytose antigens and bacteria from the intestinal lumen to antigen presenting cells on the other side. Here the authors show that the actin-binding protein Aif1 is highly expressed by intestinal M cells and regulates this transcytosis.

Intestinal tissues are continuously exposed to the outside environment. The epithelium covering the digestive tract is the barrier to invasion by gut pathogenic bacteria and interface to mutual interaction with commensal microbiota. Therefore, intestinal epithelial cells (IECs) are equipped with a variety of immunological, physiological and chemical barrier features to maintain the balance between surveillance or elimination and symbiosis, and thus create intestinal homeostasis^1^,^2^,^3^,^4^. These features include innate antigen-recognition receptors such as Toll-like receptors, along with acquired immunity (for example, in the form of secretory IgA), tight junction molecules (for example, occludin), and production of antimicrobial peptides (for example, defensin), cytokines, chemokines and mucins^4^. Offensive and defensive interactions between host and bacteria influence the induction and regulation of the antigen-specific mucosal immune responses. To induce antigen-specific immune responses against orally encountered antigens, the mucosal immune system is functionally organized into inductive tissues such as Peyer's patches (PPs) and effector tissues such as the lamina propria^5^,^6^. PPs are well-characterized inductive tissue in the small intestine and are covered by follicle-associated epithelium (FAE)^6^. FAE contains microfold (M) cells, which are specialized antigen-sampling cells that actively take up foreign antigens from the intestinal luminal side into PPs for the initiation of antigen-specific humoral and cellular immune responses^7^. M cells have two unique structural characteristics; they have irregular, short microvilli on their apical side that distinguish them from neighbouring columnar epithelial cells with tall and dense microvilli, and they have a pocket structure holding antigen-presenting cells such as macrophages, B cells, and dendritic cells on their basolateral side^8^,^9^,^10^,^11^. This unique morphology is considered to contribute to their active antigen uptake and the subsequent transcytosis of antigens from the intestinal lumen to antigen-presenting cells in PPs, resulting in the initiation of antigen-specific mucosal immune responses^7^,^12^.

Glycoprotein 2 (GP2) has been identified as a specific marker of mature M cells; it contributes to the uptake of *Salmonella enterica* serovar Typhimurium by recognising the bacterial flagellar protein FimH^13^,^14^. In addition, cellular prion protein on the M-cell surface has been reported to be an invasive receptor for *Brucella abortus*^15^. Furthermore, Spi-B is a pivotal transcription factor for the development and function of M cells, including the induction of GP2 (refs 16, 17, 18). Despite these advances, we still have a limited understanding of the molecular basis of M-cell development, maturation, and function (for example, transcytosis).

To further understand the immunobiological uniqueness of M cells, we previously performed a DNA microarray analysis to search for new molecules (our unpublished data) and identified several unique candidate genes specifically associated with M cells. From this assay, we proposed allograft inflammatory factor 1 (Aif1) as a candidate protein that is encoded by one of these genes and is preferentially produced by M cells. Aif1, which is also known as ionized calcium-binding adapter molecule 1 (Iba1), is a cytoplasmic protein with EF-hand calcium-binding domains and was first observed in the macrophages of rat heart allografts under chronic rejection^19^. Aif1 is highly expressed in the monocytic lineage, including macrophages and microglia, and is involved in phagocytosis and the formation of membrane ruffling through Rac1, which belongs to the Rho-family of GTPases^20^,^21^. In addition, when EF-hand-domain-truncated or point-mutated Aif1 is overexpressed in the microglial cell line MG5, the cells cannot form membrane ruffling through actin remodelling to catch foreign antigens such as zymosan during phagocytosis^21^,^22^. However, it has been suggested that, in vascular smooth muscle cells, Aif1-Rac1 signalling is involved in cell migration and actin polymerisation rather than membrane ruffling^23^. The precise function of Aif1 in M cells is unknown. Here we generate Aif1-deficient mice to investigate *in vivo* role of Aif1 in M cells. Aif1 deficiency does not affect the development and fundamental ultrastructure of M cells. However, uptake of particles, commensal and pathogenic bacteria by M cells is severely impaired in Aif1-deficient mice. Our findings suggest that M-cell-intrinsic Aif1 plays an important role in antigen uptake and transcytosis function of M cells.

## Results

### Specific expression of *Aif1* by M cells

To shed further light on M-cell-specific molecules, we performed a DNA microarray analysis by using RNA prepared from the FAE of *Spib*^+/+^ and *Spib*^*−/−*^ mice, because previous studies by ourselves and others had shown that Spi-B deficiency resulted in a substantial reduction in M-cell development^16^,^17^,^18^. We therefore used FAE from the *Spib*^+/+^ mice as normal FAE and that from the *Spib*^*−/−*^ mice as M-cell-deficient FAE. From this analysis we identified several candidate genes, the expression of which was identified as M-cell specific and Spi-B dependent (unpublished data). Here we focused on *Aif1*, because Aif1 is involved in phagocytosis in macrophages through modification of actin bundling^20^,^21^ and in migration of vascular smooth muscle cells through the induction of actin polymerization^23^.

**Figure 1:**
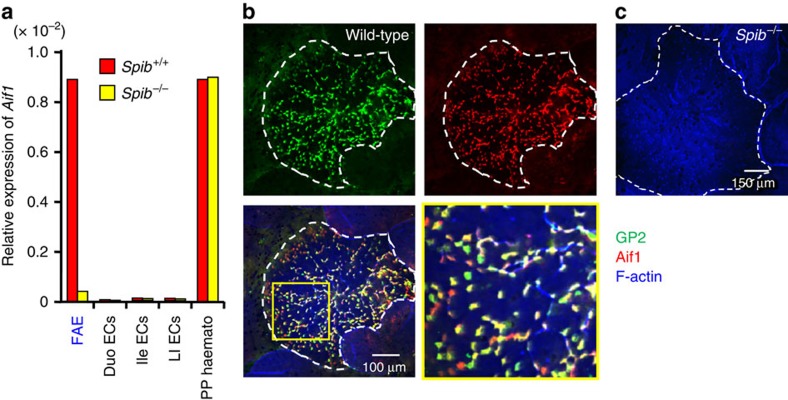
Specific expression of Aif1 in GP2-positive M cells among IECs. (**a**) Relative *Aif1* expression in indicated cell populations from *Spib*^+/+^ and *Spib*^*−/−*^ mice. Each result was normalized against the expression of *glyceraldehyde 3-phosphate dehydrogenase* (*Gapdh*) and is representative of three independent experiments. (**b**,**c**) Whole-mount staining of GP2-positive M cells and Aif1. Peyer's patches (PPs) prepared from wild-type C57BL/6J (**b**) and Spi-B-deficient mice (**c**) were stained with anti-GP2 antibody (green), anti-Aif1 antibody (red) and phalloidin (blue). The yellow box region in (**b**, bottom left) was magnified to produce the image in (**b**, bottom right). Dotted lines indicate the periphery of the FAE region. Data are representative of three independent experiments. Duo, duodenal; ECs, epithelial cells; FAE, follicle-associated epithelium, haemato, haematopoetic cells; Ile, ileum; LI, large intestine.

First, we examined M-cell-specific expression of *Aif1* by quantitative PCR analysis of various IECs, including FAE, which were isolated from Spi-B-deficient mice and littermate controls. In control mice, *Aif1* mRNA was highly expressed in haematopoietic cell lineages prepared from PPs, as reported previously (Fig. 1a)^21^. In fact, CD11c-positive cells in PPs and the lamina propria also expressed Aif1 (Supplementary Fig. 1). *Aif1* was also highly expressed in FAE, but not in other small or large intestinal epithelial cells (Fig. 1a), though its level was lower than other known M-cell markers such as *Gp2*, *Spib* and *Ccl9* (Supplementary Fig. 2). Expression of *Aif1* mRNA in FAE was severely defective in Spi-B-deficient mice. These results suggested that, among the various types of IECs, *Aif1* expression might be specific for M cells. Expression of *Aif1* in haematopoietic cells prepared from PPs was intact in Spi-B-deficient mice, further supporting the specificity of *Aif1* expression by M cells and its dependence on Spi-B (Fig. 1a).

We next used whole-mount staining of PPs to investigate the expression of Aif1 protein in M cells. PPs prepared from wild-type mice were stained with anti-Aif1 and anti-GP2 antibodies, because GP2 positivity in FAE is definitive of mature M cells^13^,^14^. In wild-type mice, although it seemed that there were some GP2- or Aif1-single positive cells because the fluorescein signals of both GP2 and Aif1 are different in each cell, most of the Aif1-positive cells located in the FAE of PPs were co-stained with anti-GP2 antibody (Fig. 1b). *Z*-stack images also showed that the expression of Aif1 was restricted in GP2-positive cells (Supplementary Fig. 3). However, Aif1 expression, like GP2 expression, was absent in the FAE of PPs prepared from *Spib*^*−/−*^ mice (Fig. 1c). On the other hand, consistent with the mRNA expression (Fig. 1a), production of Aif1 was detected in the sub-epithelial dome and lamina propria regions, even in Spi-B-deficient PPs (Supplementary Fig. 4). These data suggest that Aif1 is a newly identified M-cell-specific molecule, the expression of which depends on the presence of Spi-B.

### Generation and characterisation of Aif1-deficient mice

To investigate the functional role of Aif1 in M cells *in vivo*, we generated Aif1-deficient (*Aif1*^*−/−*^) mice (Supplementary Fig. 5a). *Aif1*^*−/−*^ mice were born in the expected Mendelian ratio and showed no developmental abnormalities. Immunoblot analysis confirmed that Aif1 expression was abolished in *Aif1*^*−/−*^ bone-marrow (BM) cells and splenocytes (Supplementary Fig. 5b). Furthermore, Aif1 expression in FAE of PPs was not detected by whole-mount staining (Supplementary Fig. 5c).

**Figure 2:**
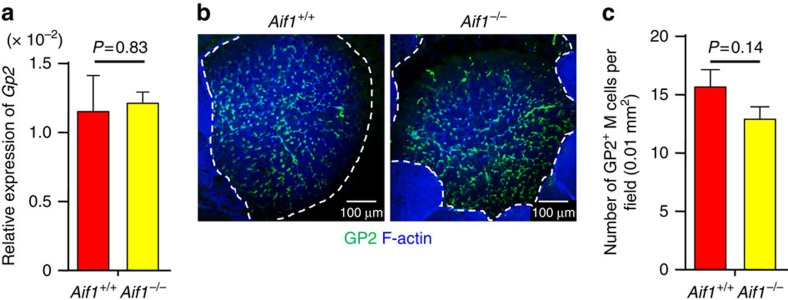
Normal M-cell numbers in Aif1-deficient mice. (**a**) Relative expression of *Gp2* in FAE from *Aif1*^+/+^ and *Aif1*^*−/−*^ mice. Each result was normalized against the expression of *Gapdh*. Data are shown as means±s.e.m. (*n*=2 per genotype) from one experiment representative of two independent experiments. *P* value was determined with Student's *t*-test. (**b**) Whole-mount staining of GP2-positive M cells in the FAE of *Aif1*^+/+^ and *Aif1*^*−/−*^ mice. The number of GP2-positive cells found in each 0.01-mm^2^ field was counted to provide the data in **c**. Dotted lines indicate the periphery of the FAE region. Data are representative of three independent experiments. (**c**) M cells were counted as GP2-positive cells, and the average number of M cells found in each 0.01 mm^2^ field is shown as the mean±s.e.m. (*n*=7 per genotype). *P* value was determined with Student's *t*-test.

Next, we checked whether there were any differences in FAE *Gp2* expression between wild-type and Aif1-deficient mice. Quantitative PCR analysis revealed that expression levels of *Gp2* mRNA in FAE were comparable in control and *Aif1*^*−/−*^ mice (Fig. 2a). In addition, almost the same number of GP2-positive cells (that is, mature M cells) per field of FAE was observed in both types of mice (Fig. 2b,c). These data suggest that Aif1 deficiency did not affect M-cell maturation.

### M-cell morphology is unaffected by Aif1-deficiency

**Figure 3:**
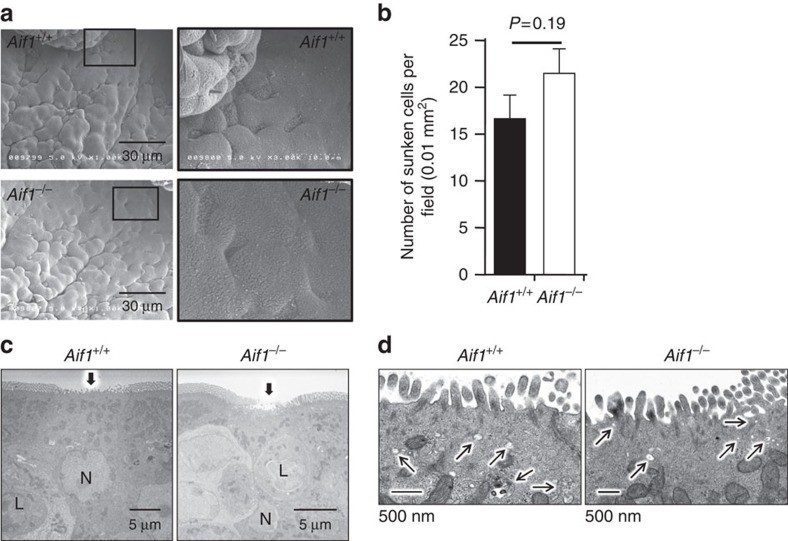
Ultrastructure of M cells in Aif1-deficient mice. (**a**) Scanning electron microscopic images of FAE from control and *Aif1*^*−/−*^mice. Right panels are magnified images of the boxed regions in the left panels. (**b**) M cells were counted as sunken cells, and the average number of M cells found in each 0.01 mm^2^ field is shown as the mean±s.e.m. of one experiment representative of two independent experiments (*Aif1*^+/+^, *n*=9; *Aif1*^*−/−*^, *n*=10). *P* value was determined with Student's *t*-test. (**c**) Transmission electron microscopic images of FAE from control and *Aif1*^*−/−*^mice. Arrows indicate M cells. Data are representative of three independent experiments. N, nucleus; L, lymphocyte. (**d**) Magnified transmission electron microscopic images of the apical side of M cells. Arrows show endosomes. Data are representative of three independent experiments.

As indicated above, Aif1 deficiency did not influence the production of GP2 by M cells. In our next experiment, we examined whether Aif1 was involved in M-cell morphology. Scanning electron microscopic analysis revealed that the apical surface morphology (short, irregular microvilli formation) of M cells in *Aif1*^*−/−*^ mice was similar to that in control mice (Fig. 3a). Consistent with the findings on GP2 positivity, the number of indented cells (which are considered to be M cells^16^,^24^) per field of FAE in *Aif1*^*−/−*^ mice did not differ significantly from that in control mice (Fig. 3b). Furthermore, the presence of microvilli and a pocket structure enfolding lymphocytes was observed in the FAE region of Aif1-deficient mice, in the same way as in wild-type mice (Fig. 3c). In addition, the endocytic compartments found at the apical side of M cells were observed in *Aif1*^*−/−*^ mice, although the size and number of endosomes were slightly smaller than those found in control mice (Fig. 3d). These data suggest that Aif1 is not associated with the morphology of M cells.

### Aif1 is important for antigen-uptake function of M cells

**Figure 4:**
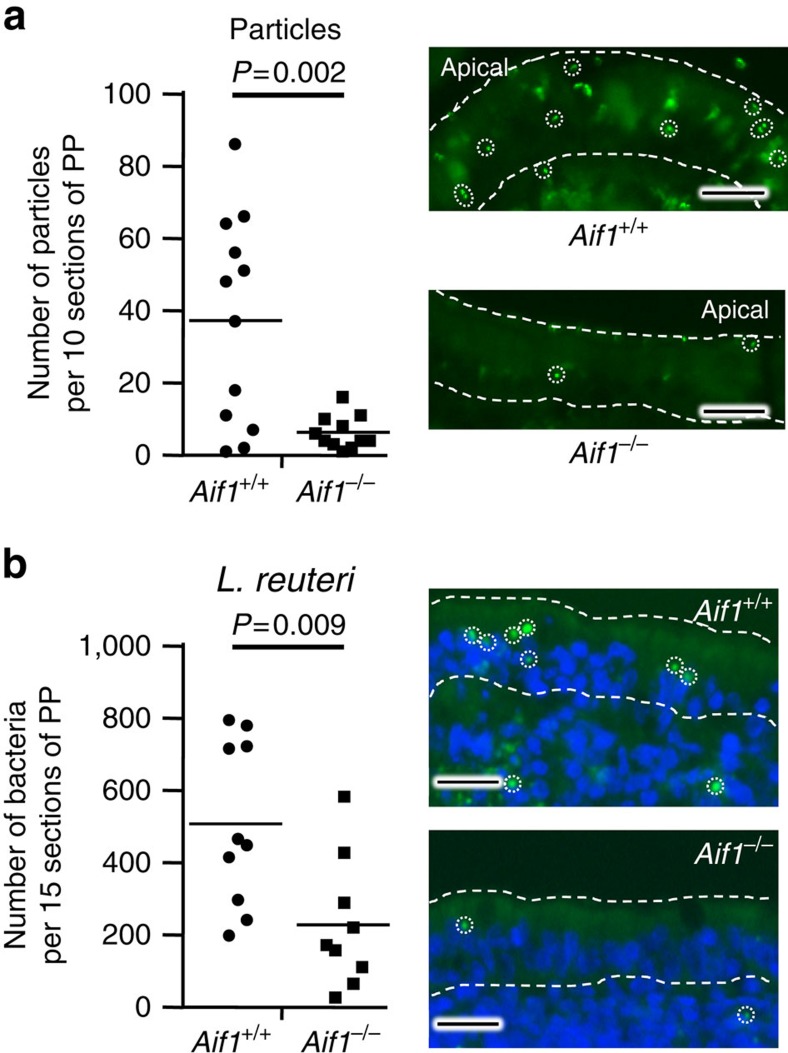
Influence of Aif1 deficiency on transcytosis function of M cells. Evaluation of particle (**a**) and *Lactobacillus reuteri* (**b**) sampling by M cells. Mice fasted for 24 h were given fluorescence-conjugated 200 nm particles (1 × 10^11^ beads) or FITC-labelled bacteria (5 × 10^9^ CFUs) orally. Two hours later, PPs were collected from the duodenum, fixed in 4% paraformaldehyde, and cut into 10 or 15 consecutive sections. The numbers of particles or labelled bacteria that had entered each PP were then counted under a fluorescence microscope. Each dot represents an individual PP from three independent experiments (For particles: *Aif1*^+/+^, *n*=12; *Aif1*^*−/−*^, *n*=11. For *L. reuteri*: *Aif1*^+/+^, *n*=10; *Aif1*^*−/−*^, *n*=9). Horizontal bars indicate each mean. *P* values were determined with Student's *t*-test. Right panels are representative images of each section. Dotted lines indicate FAE regions and circles indicate each particle or bacterium. Scale bar, 20 μm.

Although Spi-B has been shown to be a key transcription factor associated with M-cell development^16^,^17^,^18^, very little is known about the molecular mechanisms of the M-cell's antigen-sampling system. We therefore next examined whether Aif1 contributed to the antigen-uptake function of M cells. First, we compared the uptake of fluorescent particles (200 nm in diameter) between control and *Aif1*^*−/−*^ mice. Two hours after oral administration of these particles, PPs from the duodenum were removed and the number of particles in each PP was counted under a fluorescence microscope. The PPs of *Aif1*^*−/−*^ mice took up dramatically and significantly fewer particles than did the PPs of *Aif1*^*+/+*^ mice (Fig. 4a). We then performed the same assay by using fluorescence-labelled *Lactobacillus reuteri* as larger particulate antigens (0.7 to 1.0 × 2.0 to 3.0 μm in size, ref. 25); this bacterium is one of the major commensals found in the mouse small intestine^26^. The numbers of bacteria found in FAE and inside the PPs were significantly smaller in *Aif1*^*−/−*^ mice than in control *Aif1*^+/+^ mice (Fig. 4b).

**Figure 5:**
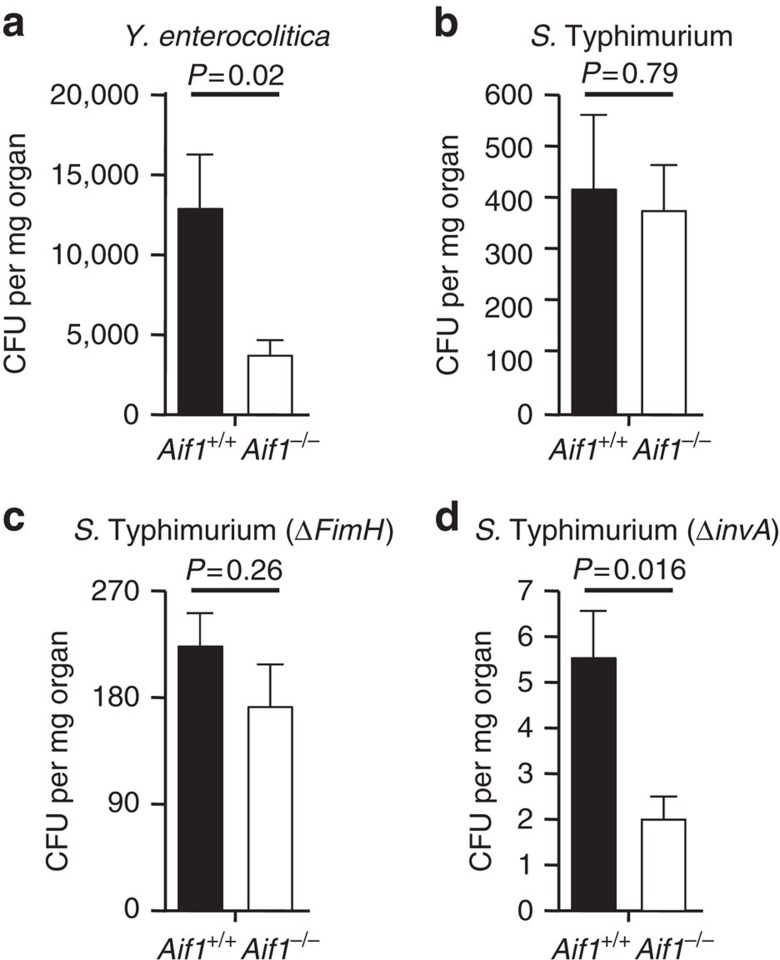
Impaired translocation of pathogenic bacteria in *Aif1*^*−/−*^ mice. Evaluation of transcytosis of *Y. enterocolitica* (**a**; 1 × 10^9^ CFUs given orally) and indicated strains of *S.* Typhimurium (**b**–**d**; 1 × 10^8^ CFUs given orally). Twenty-four hours after administration of the bacteria, PPs were collected from the ileum, homogenized and diluted. Each diluted sample was cultured on the appropriate plates. Colonies were counted and the values normalized against the weight of the PPs. Data are shown as means±s.e.m. from two independent experiments (*n*=18 per genotype for *Y. enterocolitica*; *n*=10 to 20 per genotype for *S.* Typhimurium). *P* values were determined with Student's *t*-test.

Next, we used *Yersinia enterocolitica* and *S.* Typhimurium to examine the influence of Aif1 deficiency on M-cell-targeted invasion of PPs by intestinal pathogenic bacteria. Wild-type control and *Aif1*^*−/−*^ mice were orally given 1 × 10^9^ colony-forming units (CFUs) of *Y. enterocolitica* or 1 × 10^8^ CFUs of *S.* Typhimurium. Twenty-four hours after oral administration of the pathogens, PPs were isolated from the ileum, weighed, homogenized, and diluted in PBS. Each diluted sample was plated on *Y. enterocolitica*-selective agar, or on LB agar plates containing 25 μg ml^−1^ nalidixic acid to select *S.* Typhimurium, before overnight incubation at the appropriate temperatures (see Methods section). We then counted the numbers of colonies. Surprisingly, in a result similar to that for the particle uptake, the number of CFUs of *Y. enterocolitica* found in the PPs was significantly lower in *Aif1*^*−/−*^ mice than in wild-type mice (Fig. 5a). In contrast, the number of *S.* Typhimurium found in the PPs did not differ significantly between control and *Aif1*^*−/−*^ mice (Fig. 5b).

As shown above (Fig. 2b), the expression of GP2 was not impaired in Aif1-deficient mice. Given that GP2 recognizes the outer membrane protein FimH on *S.* Typhimurium and contributes to efficient transcytosis of such bacteria by M cells^14^, the presence of GP2 at the apical surfaces of M cells upon uptake of *S.* Typhimurium might be the reason why Aif1-deficient mice showed no decline in the number of *S.* Typhimurium in the PPs. To investigate this hypothesis, we next prepared and used a *FimH*-deletion mutant of *S.* Typhimurium (Δ*FimH*). In wild-type mice, the number of Δ*FimH S.* Typhimurium entering the PPs was smaller than the number of parental *FimH*-positive bacteria entering (Fig. 5c,b). Translocation of Δ*FimH S.* Typhimurium compared with parental *FimH*-positive bacteria was also diminished in *Aif1*^*−/−*^ mice, but contrary to our speculation, this effect was no more than in wild-type mice (Fig. 5c). *Salmonella* spp. invasion induces membrane ruffling by their type-three secretion system (TTSS)^27^. In the case of *Yersinia* spp. invasion, membrane ruffling is not needed and does not occur^27^,^28^. Therefore, we next examined the uptake of the Δ*invA* mutant of *S.* Typhimurium, which lacks TTSS and cannot induce membrane ruffling^27^. Because of this lack of TTSS, the uptake number of Δ*invA* mutant organisms was severely reduced, even in wild-type control mice, but it was more severely reduced in *Aif1*^*−/−*^ mice (Fig. 5d; *P*=0.016 versus wild-type mice). Taken together, these results suggest that Aif1 is associated with actin remodelling occurring on *Y. enterocolitica* invasion, but not with membrane ruffling, which is induced by, and needed for, invasion of *S.* Typhimurium.

### M-cell-intrinsic Aif1 is important for mucosal immunity

**Figure 6:**
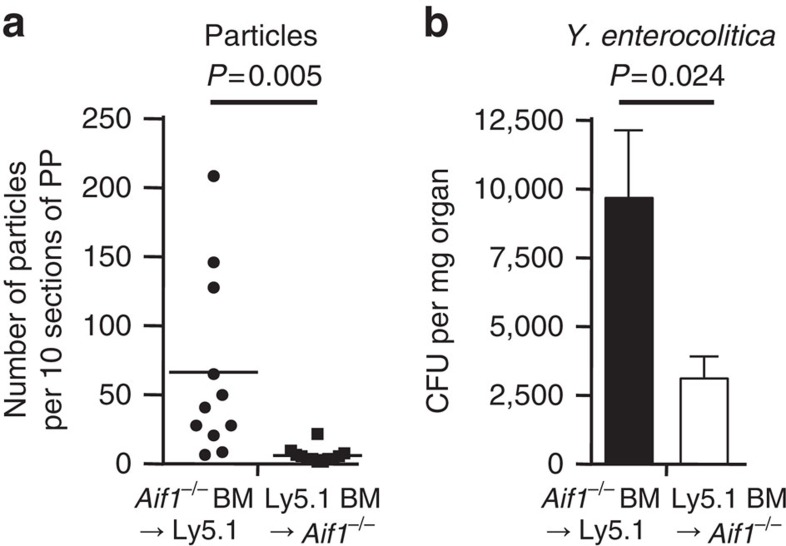
Importance of M-cell-intrinsic Aif1 for uptake of particles and *Y. enterocolitica.* Evaluation of 200 nm bead uptake (**a**) and transcytosis of *Y. enterocolitica* (**b**) in bone-marrow (BM) chimeric mice to which we transferred *Aif1*^*−/−*^ BM cells (*Aif1*^*−/−*^ BM→Ly5.1) or vice versa (Ly5.1 BM→*Aif1*^*−/−*^). Both experiments used the same protocols as described in Fig. 4a (particles) and Fig. 5a (*Y. enterocolitica*; for particles: *n*=12 per group. For *Y. enterocolitica*: *Aif1*^*−/−*^ BM→Ly5.1, *n*=5; Ly5.1 BM→*Aif1*^*−/−*^, *n*=6). *P* values were determined with Student's *t*-test.

Aif1-deficient mice showed decreased M-cell-mediated uptake of particles and translocation of *Y. enterocolitica*. Aif1 expression has been reported so far in microglia and macrophages^21^,^29^. Therefore, it was also important to investigate whether Aif1, which is produced in macrophages, dendritic cells, and lymphocytes, contributed to the uptake of particles and *Y. enterocolitica*. We used BM chimeric mice prepared by transplanting *Aif1*^*−/−*^ BM cells into congenic wild-type (Ly5.1) mice (*Aif1*^*−/−*^ BM→Ly5.1) or vice versa (Ly5.1 BM→*Aif1*^*−/−*^). Both types of chimeric mice were orally given the microparticles (200 nm in diameter) or *Y. enterocolitica* 8 weeks after BM transfer. Uptake of either particles or *Y. enterocolitica* was not influenced by Aif1-deficient BM transfer in Ly5.1 mice (Fig. 6a,b). Transfer of wild-type BM to *Aif1*^*−/−*^ mice did not improve the significantly reduced particle or *Y. enterocolitica* uptake in these mice compared with that in the *Aif1*^*−/−*^ BM→Ly5.1 mice. These results suggest that Aif1 expression in M cells, but not in haematopoietic cells, contributes to the uptake of particles and *Y. enterocolitica*.

**Figure 7:**
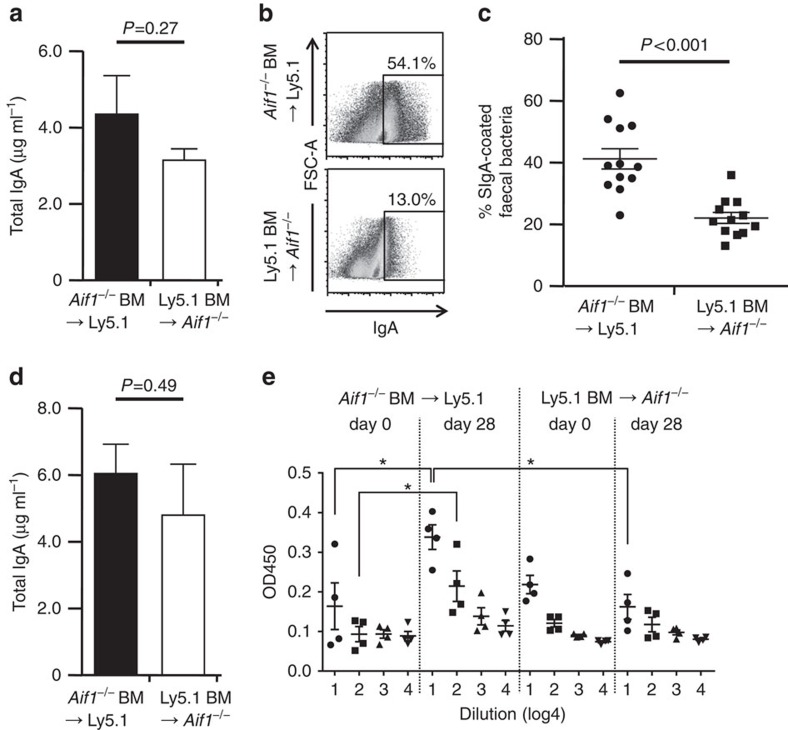
Importance of Aif1 for initiation of the mucosal IgA immune response. (**a**) Quantification of total faecal IgA. Faeces were prepared from the chimeric mice indicated. Concentrations of total IgA were determined by ELISA. Data are means±s.e.m. from two independent experiments (*n*=8 per group). *P* value was determined with Student's *t*-test. (**b**) Representative plots of bacterial flow cytometry. Faeces were collected from the chimeric mice indicated and suspended in PBS. Bacteria recognized by IgA were detected and their percentages determined. (**c**) The percentages determined in **b** are shown as means±s.e.m. from two independent experiments (*n*=12 per group). *P* value was determined with Student's *t*-test. (**d**) Quantification of total IgA after long-term infection with *Y. enterocolitica*. Faeces were prepared from the indicated chimeric mice on day 28 after infection. Concentrations of total IgA were determined by ELISA. Data are means±s.e.m. from two independent experiments (*n*=6 per group). *P* value was determined with Student's *t*-test. (**e**) Comparison of anti-*Y. enterocolitica-*specific IgA production after long-term infection. Serial diluted faecal extracts prepared from *Y. enterocolitica-*infected mice were subjected to ELISA by using *Y. enterocolitica-*coated plates. Data are means±s.e.m. from one experiment representative of two independent experiments (*n*=4 per group). *P<0.05, determined with Student's *t*-test.

Aif1-deficient mice showed impaired uptake of the major enteric commensal bacterium, *L. reuteri* (Fig. 4b). Given that M-cell-intrinsic Aif1 is important for the uptake of substantial numbers of commensal bacteria, it is possible that secretory IgA (SIgA) production is also diminished in Ly5.1 BM→*Aif1*^*−/−*^ chimeric mice, because IEC-specific RANK-deficient mice, which lack PP M cells, have less faecal SIgA than do control mice^30^. Enzyme-linked immunosorbent assay (ELISA) examination of the amounts of faecal total IgA in both types of Aif1 chimeric mice revealed no significant difference between the two groups (Fig. 7a), suggesting that entry of commensal bacteria was not totally impaired in Ly5.1 BM→*Aif1*^*−/−*^ chimeric mice. Interestingly, however, the population of IgA-coated faecal bacteria was significantly reduced in Ly5.1 BM→*Aif1*^*−/−*^ chimeric mice compared with that in *Aif1*^*−/−*^ BM→Ly5.1 mice (Fig. 7b,c).

Next, we measured anti-*Y. enterocolitica-*specific antibody in a model of long-term infection. *Y. enterocolitica* at 1 × 10^8^ CFUs was given orally to the chimeric mice once a week to analyse total and antigen-specific IgA antibody induction. After four inoculations of *Y. enterocolitica*, Ly5.1 recipient mice, which possessed wild-type M cells and Aif1-deficient haematopoietic cells, produced large amounts of total and anti-*Y. enterocolitica* faecal IgA antibodies (Fig. 7d,e). Although *Aif1*^*−/−*^ recipient mice, which possessed Aif1-deficient M cells and wild-type haematopoietic cells, had comparable levels of total IgA in their faeces after infection as before infection (Fig. 7a,d), no induction of anti-*Y. enterocolitica-*specific IgA production was observed in these mice. These data strongly suggest that M-cell-intrinsic Aif1 expression is important to the translocation of *Y. enterocolitica*, and that this translocation was followed by the induction of a mucosal IgA immune response to this bacterium.

### Aif1-dependent activation of β1 integrin on M-cell surface

**Figure 8:**
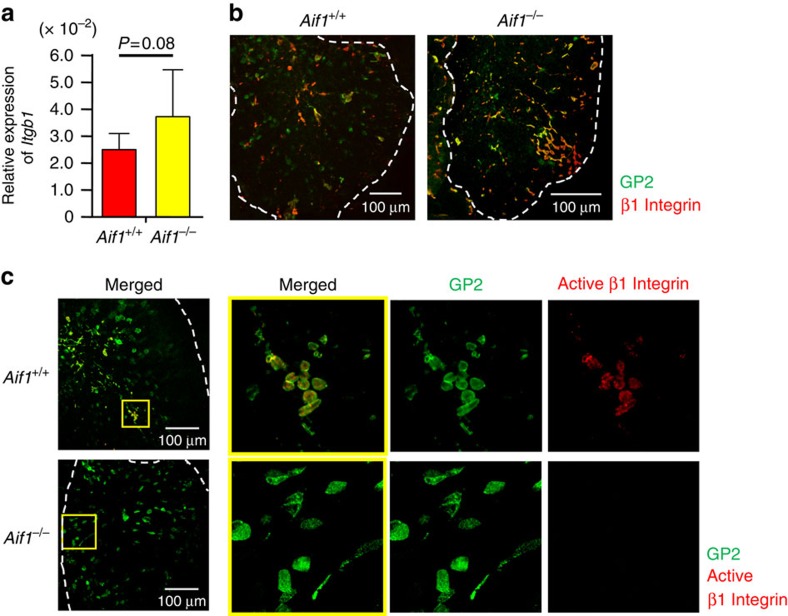
Aif1-dependent activation of β1 integrin. (**a**) Relative gene expression of β1 integrin in FAE from wild-type and *Aif1*^*−/−*^ mice. Data are means±s.e.m. of one experiment representative of two independent experiments (*n*=4 per genotype). *P* value was determined with Student's *t*-test. (**b**,**c**) Whole-mount staining of PPs prepared from *Aif1*^+/+^ and *Aif1*^*−/−*^ mice with anti-total β1 integrin antibody (**b**, red) or anti-activated-β1 integrin antibody (**c**, red), together with anti-GP2 antibody (**b**,**c**, green). Each yellow box region in **c** (left panels) was magnified to produce the image in **c** (right panels). Dotted lines indicate the periphery of the FAE region. Data are representative of three independent experiments.

Invasin, which is one of the outer proteins of *Y. enterocolitica*, is a specific ligand molecule that binds to β1 integrin on the apical surfaces of M cells to facilitate effective entry of *Y. enterocolitica* into PPs^31^,^32^,^33^,^34^. We hypothesized that apical surface expression or activation, or both, of β1 integrin in M cells would be regulated by Aif1 and would thus be diminished in Aif1-deficient mice. However, the level of β1 integrin mRNA (*Itgb1*) expression did not differ significantly between FAEs prepared from control and *Aif1*^*−/−*^mice (Fig. 8a). Next, we examined the surface expression of total and activated β1 integrin on the apical side of M cells by using whole-mount staining without cell permeabilization. We examined the apical side because non-M-cell enterocytes also express β1-integrin on the lateral membranes, but not on the apical surfaces^34^. Total β1 integrin expression was detected on the apical surfaces of GP2-positive M cells in control and *Aif1*^*−/−*^mice (Fig. 8b), whereas active β1 integrin expression on the apical surfaces of M cells was seen in control mice but not in Aif1-deficient mice (Fig. 8c). These data suggested that Aif1 was important for the activation of β1 integrin on the apical surfaces of M cells contributing to the translocation of *Y. enterocolitica* via invasin.

## Discussion

Here we identified Aif1 as a novel molecule specifically expressed in M cells among IECs, and we newly generated Aif1-deficient mice for *in vivo* elucidation of the role of Aif1 in M-cell development and function. We demonstrated that Aif1 is important for the M-cell uptake function of extraneous substances such as microparticles and certain commensal bacteria, including *L. reuteri*, but that it is not involved in the structural development of M cells. Furthermore, Aif1 is involved in the activation of β1 integrin, which is a potential receptor for the invasin of *Y. enterocolitica*, at the apical surface of M cells for the effective entry of this pathogenic bacterium into PPs and the subsequent induction of antigen-specific IgA immune responses.

Because Aif1 is also strongly expressed in the testis, it has been hypothesized that Aif1 has a role in spermatogenesis^19^,^35^. However, our strain, and another strain, of Aif1-deficient male mice showed normal fertility (data not shown and ref. 36). Aif1 expression has also been found in another epithelial cell, namely the glomerular visceral epithelial cell, or podocyte, which partially covers the outside of the glomerular basement membrane and acts as a final barrier to protein loss in the kidney^37^. The function of Aif1 in podocytes is unknown; however, it very likely has a role related to the transport of materials, because it is expressed in epithelial cells specialized for substance transfer or filtration, such as the M cells in IECs.

Aif1 expression in M cells was totally dependent on Spi-B, which is a master regulator of M-cell development^16^,^17^,^18^. Interestingly, however, gene expression and protein production of Aif1 in the haematopoietic cells in PPs were not affected by Spi-B deficiency (Fig. 1a; Supplementary Fig. 4). Recently, it has been reported that expression of *Aif1* in microglia is dependent on interferon regulatory factor 8 (IRF8)^38^,^39^. In addition, expression of Aif1 in microglial cells and IFNγ-stimulated macrophages requires the transcription factor PU.1 (refs 40, 41). In fact, in the predicted promoter region of *Aif1* there are several consensus binding sites for transcription factors, including those of the Ets, IRF and STAT families^40^. Because PU.1 belongs to the Ets family and, among the members of that family, is closest to Spi-B, it is possible that Aif1 is a direct target of Spi-B in M cells. Another explanation for the regulation of *Aif1* is that another transcription factor—perhaps PU.1 or IRF8—is upregulated or activated, or both, in M cells to trigger the transcription of *Aif1*.

Numerous *in vitro* experiments have indicated that Aif1 is involved in actin polymerization in inflammation^42^,^43^. In fact, in a collagen-induced arthritis model, Aif1-deficient mice showed attenuated signs of arthritis^36^. There have been also many reports that Aif1 is involved in actin remodelling, ruffling and bundling as part of the phagocytic function of macrophages and microglial cells^20^,^21^,^22^,^29^ and as part of the migration of vascular smooth muscle cells^23^. Therefore, we hypothesized that Aif1 deficiency would affect the ultrastructure of M cells. However, a sunken structure, which is one of the features of the apical surfaces of M cells^7^,^12^, was easily and consistently detected in our Aif1-deficient mice (Fig. 3a,b). Transmission electron microscopic analysis also revealed that Aif1-deficient mice possessed cells with M-cell morphology in their FAE, which had irregular microvilli and endosomes on the apical side and a pocket structure on the basolateral side (Fig. 3c,d). Furthermore, whole-mount staining showed that the numbers of GP2-positive cells found in FAE were almost the same in wild-type and Aif1-deficient mice (Fig. 2b,c). Interestingly, although Aif1 is presumably a cytoplasmic molecule, *Z*-stacked transverse images from whole-mount staining showed that localisation of Aif1 was abundant on the apical side, but not the basolateral side, of M cells (Supplementary Fig. 3). These data suggest that Aif1 is not involved in actin remodelling on the basal side, nor in the fundamental structural morphology or maturation of M cells.

Our study also demonstrated that uptake of particles and *L. reuteri* by M cells was significantly less in *Aif1*^*−/−*^ mice than in wild-type mice (Fig. 4a,b). These results suggest that Aif1 plays a critical role in M-cell-mediated antigen uptake from the intestinal lumen—a unique characteristic of these cells. It remains unknown precisely what stimuli activate Aif1; however, it is interesting to postulate that Aif1 acts when transcytosis has just occurred at the apical surface of the M cell, because Aif1 deficiency seemed not to affect cell-structural morphology. In relation to this issue, although the percentage of SIgA-coated faecal bacteria was significantly lower in Ly5.1 BM→*Aif1*^*−/−*^ chimeric mice than in *Aif1*^*−/−*^ BM→Ly5.1 mice, certain bacteria were still recognized by SIgA in the Ly5.1 BM→*Aif1*^*−/−*^ mice (Fig. 7b,c), because no IgA-coated bacteria were observed in faeces prepared from Rag1-deficient mice, which lack T and B cells. Taken together with the evidence that total IgA production levels were comparable in the above mentioned two types of chimeric mice (Fig. 7a), these results suggest that Aif1-independent uptake of some kinds of commensal bacteria occurs continuously at M cells. This might also explain why endosomes were still observed in Aif1-deficient M cells (Fig. 3d). One possible mechanism of Aif1-independent uptake is IgA-mediated transcytosis by Dectin-1 because it has recently been reported that Dectin-1 acts on IgA-mediated transcytosis in M-like cells derived from Caco-2 cells, a human colonic cancer cell line, and mouse M cells^44^. However, we considered that Dectin-1 is not contributed on SIgA-mediate antigen uptake on M cells because the *Dectin-1* mRNA level in the FAE of mouse PPs was very low (data not shown). In addition, we could not detect Dectin-1-positive cells in mouse PP FAE by whole-mount staining using anti-mouse Dectin-1 mAb (clone: RH1; Supplementary Fig. 6), indicating that Dectin-1 is not expressed on the apical side of M cells. Furthermore, *Dectin-1* has never been identified from several gene-expression and profiling data sets as an FAE- or M-cell-specific gene.

In addition to the defects in uptake of particles and some kinds of commensal bacteria in Aif1-deficient mice, invasion of M cells by *Y. enterocolitica* was also significantly decreased in these mice, whereas uptake of *S.* Typhimurium was normal (Fig. 5a,b). Upon the entry of pathogenic bacteria into host epithelial cells, reorganisation of the actin cytoskeleton is induced by ‘zipper' or ‘trigger' mechanisms^27^,^28^. The zipper mechanism, which is induced upon invasion by *Yersinia*, *Listeria* and *Neisseria* spp., is initiated by bacterial ligand and host cellular receptor interaction. On the other hand, the trigger model is observed upon entry of *Salmonella* and *Shigella* spp., with the induction of membrane ruffling. Given that Aif1 is associated with the activation of β1 integrin, but not with the expression of GP2, in M cells (Fig. 8b,c and 2b), it is possible that actin rearrangement at the apical side of M cells is not induced by *Y. enterocolitica* in Aif1-deficient mice, because β1 integrin acts as a receptor for the invasin molecules of *Yersinia* spp.^31^,^32^,^33^,^34^. Consistently, uptake of Δ*invA S.* Typhimurium, which did not induce membrane ruffling, was significantly lower in Aif1-deficient mice than in control mice (Fig. 5d).

Our data strongly indicate that M-cell-intrinsic Aif1 is important for not only uptake of particles, some kinds of commensal bacteria, and *Y. enterocolitica*, but also for the subsequent activation of mucosal IgA immune responses. Because CD11c-positive cells in PPs and the lamina propria also expressed Aif1 (Supplementary Fig. 1), Aif1 might contribute to antigen presentation by antigen-presenting cells or to antigen sampling by CD11c^+^CX_3_CR1^+^ dendritic cells, which are found in the sub-epithelial dome region of PPs and can directly sample bacteria from the intestinal lumen^45^,^46^. However, this possibility was contradicted, because the wild-type mice to which we adoptively transferred *Aif1*^*−/−*^ haematopoietic cells (*Aif1*^*−/−*^ BM→Ly5.1: mice harbouring normal M cells and *Aif1*^*−/−*^ antigen-presenting cells) showed induction of antigen-specific SIgA production, whereas this was severely defective in our other chimeric mice (Ly5.1 BM→*Aif1*^*−/−*^: mice harbouring *Aif1*^*−/−*^ M cells and normal antigen-presenting cells) after *Y. enterocolitica* infection (Fig. 7e).

Small GTPases contribute to the activation of integrins via Ca^2+^-binding molecules such as calmodulin^47^. Because Aif1 has Ca^2+^-binding EF hand motifs, and because we previously reported that *Rac2* is specifically expressed in M cells among IEC lineages^13^, it is possible that Rac2 mediates Aif1-dependent β1 integrin activation in M cells. In fact, Aif1-mediated activation of Rac2 has been shown to be important for the activation of vascular smooth muscle cells^48^. Taken together with the data shown in this study, it can be suggested that Aif1 expression in M cells contributes to the activation of Rac2, leading to (1) activation of β1 integrin, which contributes to the translocation of *Y. enterocolitica*, and (2) actin remodelling, which contributes to the uptake of particle antigens and enteric commensal bacteria by M cells, though future studies are required to elucidate further the mechanisms of how Aif1 controls actin remodelling and the activation of β1 integrin *in vivo*.

## Methods

### Generation of Aif1-deficient mice

A mouse *Aif1* genome clone (GenBank Accession # AB036423) was isolated by using a mouse *Aif1* cDNA probe to screen a 129 SVJ mouse genome cosmid library (Stratagene). The targeting vector was constructed on pBluescriptIISK(-) (Stratagene) by inserting a diphtheria toxin A (DT-A) expression cassette, a KpnI–HindIII fragment containing *Aif1* exons 1 and 2 and part of exon 3, a neomycin resistance cassette, a HindIII–NotI fragment corresponding to the 3′-downstream region of *Aif1*, and another DT-A cassette (Supplementary Fig. 5a). The HindIII site of the 3′-fragment was destroyed by ligation with the neomycin cassette. The vector was linearized by digestion with XhoI and electroporated into embryonic stem cells. Embryonic stem cell clones selected by G418 resistance were screened for homologous recombination by Southern blot analysis (data not shown). These clones were individually microinjected into blastocysts derived from C57BL/6 mice and transferred to pseudopregnant females. A chimeric female mouse was mated with a 129 male mouse; their offspring, which were judged as *Aif1* heterologously deleted (*Aif1*^+/−^), were then backcrossed for more than eight generations with C57BL/6J mice. Heterozygous mice were crossed to obtain *Aif1*^+/+^ and *Aif1*^*−/−*^ mice. Littermates or age- and gender-matched *Aif1*^+/+^ and *Aif1*^*−/−*^ mice were used for all subsequent experiments in this study. *Aif1* genotyping was performed by PCR method using the sets of primers: for wild-type allele, 5′-GGCTTCAAGTTTGGACGGCAGATCCTC-3′ and 5′-CATGAGCCAAAGCAGGGATTTGCAGGG-3′; and for mutated allele, 5′-ATGAGCCAAAGCAGGGATTTG-3′ and 5′-CGACCTGCAGCCAATATGGG-3′.

### Mice

*Spib*^*−/−*^ mice (C57BL/6 background) were kindly provided by Dr M. Celeste Simon (University of Pennsylvania). CD45.1 congenic mice on the C57BL/6 background (Ly5.1) and *Rag1*^*−/−*^ mice on the C57BL/6 background were purchased from Jackson Laboratory. C57BL/6J mice were obtained from CLEA Japan. Until use, all mice were maintained under specific-pathogen-free conditions in horizontal laminar flow cabinets in the experimental animal facility of the Institute of Medical Science, The University of Tokyo. Six to 12 weeks old mice of both genders were used in this study. All animal experiments were conducted with the approval of the Animal Research Committee of the Institute of Medical Science, The University of Tokyo.

### Real-time PCR

To isolate and prepare FAE from PPs, dissected PPs were washed and then incubated in PBS containing 30 mM EDTA and 1 mM DTT for 30 min on ice. The FAE was peeled off by manipulation with fine needles under a stereoscopic microscope. Total RNA was prepared from the cells by using an RNeasy Kit (Qiagen). Complementary DNA was reverse transcribed by using SuperScript VILO master mix (Invitrogen), and then used as a template for real-time PCR with a set of specific primers and a LightCycler 480 SYBR Green I Master (Roche) on a LightCycler 480 System II (Roche). Expression levels of target genes were normalized against that of *glyceraldehyde 3-phosphate dehydrogenase* (*Gapdh*). The sets of primers used were as follows: *Gapdh* (forward) 5′-TGTCCGTCGTGGATCTGAC-3′ and (reverse) 5′-CCTGCTTCACCACCTTCTTG-3′; *Aif1* (forward) 5′-GGATTTGCAGGGAGGAAAA-3′ and (reverse) 5′-TGGGATCATCGAGGAATTG-3′; *Gp2* (forward) 5′-ATACTGCACAGACCCCTCCA-3′ and (reverse) 5′-GCAAACTCTGAGAGTCAGAAACATG-3′; *Spib* (forward) 5′-GCCCACACTTAAGCTGTTTGTA-3′ and (reverse) 5′-CTGTCCAGCCCCATGTAGAG-3′; and *Itgb1* (forward) 5′-ctgcttctaaaattgagatcagga-3′ and (reverse) 5′-tccataaggtagtagagatcaataggg-3′.

### Electron microscopic analysis

PPs dissected from the ileum were fixed at room temperature for 2 h in a solution containing 2.5% glutaraldehyde, 2% paraformaldehyde and 0.1 M phosphate buffer (pH 7.4). After being washed with 3% sucrose three times on ice, the samples were fixed in 1% osmium tetraoxide on ice for 1 h and dehydrated sequentially with 30, 50, 70 and 90% ethanol for 15 min each. For scanning electron microscopy, dehydrated samples were freeze-embedded in *t*-butyl alcohol and freeze dried, and then coated with osmium. Each sample was then observed under a scanning electron microscope (S-4200; Hitachi). For transmission electron microscopy, dehydrated samples were treated with propylene oxide twice for 5 min, and then embedded in Epon812 resin mixture (TAAB Laboratories). Ultra-thin sections (70 nm) stained by 2% uranyl acetate and Reynolds lead solution were examined under a Hitachi H-7500 electron microscopy.

### Whole-mount staining

PPs were excised from the duodenum and ileum and then washed with PBS. The PPs were then fixed, permeabilized, and blocked with a BD Cytofix/Cytoperm kit (BD Biosciences) or just fixed by 4% paraformaldehyde and blocked with 0.2% BSA/PBS. Permeabilized tissues were stained with rat anti-mouse GP2 antibody (MBL) and rabbit anti-mouse Aif1 antibody (Wako), followed by the appropriate secondary antibodies conjugated with Alexa Fluor 488 or Cy3 (Jackson ImmunoResearch). After being washed with PBS, the specimens were stained with Alexa Fluor 633-conjugated Phalloidin (Invitrogen). Unpermeabilized tissues were stained with rat anti-mouse integrin beta 1 (clone 265917, R&D) or rat anti-mouse active integrin beta 1 (clone 9EG7, BD Biosciences), followed by Cy3-conjugated secondary anti-rat Ig antibody. After being washed, samples were then stained with Alexa Fluor 488-conjugated anti-mouse GP2 antibody (MBL). Samples were mounted and analysed under a confocal laser-scanning microscope (DM IRE2/TCS SP2; Leica or FV1200; Olympus).

### Quantification of antigen uptake by M cells

About 1 × 10^11^ particles (200 nm in diameter; Yellow Green Microspheres, Polysciences) or FITC-labelled *L*. *reuteri* (5 × 10^9^ CFUs) were given orally to mice that had been fasted for 24 h. Two hours later, two PPs were dissected from the duodenum of each mouse and embedded in OCT compound. After we had made 10 or 15 successive frozen sections, the particles or bacteria found inside the PPs were observed and counted under a fluorescence microscope (Keyence).

### Bacteria

*Lactobacillus reuteri* (ATCC 23272) obtained from RIKEN BioResource Center were grown overnight at 37 °C under anaerobic conditions while left standing in MRS broth (BD Biosciences). For FITC labelling, 5 × 10^9^ *L. reuteri* were incubated with 5 mg ml^−1^ Fluorescein isothiocyanate isomer I–Celite (Sigma) at 37 °C for 60 min with shaking. Nalidixic acid-resistant *S*. Typhimurium (strain *χ*3306)^49^ and *invA*-deletion mutants of *χ*3306 (strain UF102)^50^, which possess a kanamycin resistance gene, were kindly provided by Dr Hidenori Matsui of Kitasato University. To generate *FimH*-deletion mutants of *S*. Typhimurium, pKD46 plasmid encoding Red recombinase^51^ was introduced into *χ*3306. *χ*3306 carrying pKD46 were then transformed with a PCR fragment containing the kanamycin resistance cassette and regions homologous to the *FimH* gene; the fragment was prepared with pKD4 plasmid as a template and the primer set of 5′-CAACCTGTATCCGTCCGGCGTCATAAAAGGAAAAATAGAGGTGTAGGCTGGAGCTGCTTC-3′ and 5′-CGATAGCGATGAAAACGCGCCGAAGGATCATTATGCCTCCATGGGAATTAGCCATGGTCC-3′. After culture of the above transformants in LB broth containing L-arabinose and kanamycin, ampicillin-sensitive clones that had lost the pKD46 were selected and then checked for *FimH* deletion by PCR. All *S*. Typhimurium strains were grown overnight at 37 °C in LB broth containing nalidixic acid (and kanamycin for strain UF102 and Δ*FimH*). *Y. enterocolitica* (ATCC 27729) was grown overnight at 26 °C in tryptic soy broth. Following overnight incubation, a culture of *S*. Typhimurium and one of *Y. enterocolitica* were prepared such that the OD_600_ reached 0.1 to 0.2; they were placed into fresh growth medium and further incubated for 2 h at the appropriate temperatures for each, as above.

### Bacterial administration assay

*Aif1*^*+/+*^ and *Aif1*^*−/−*^ mice were each given 100 μl of 1 × 10^9^ CFU per ml *S*. Typhimurium or 1 × 10^10^ CFU per ml *Y. enterocolitica* by mouth. After 24 h, PPs were dissected from the ileum, washed with PBS, and then incubated in 100 μg ml^−1^ gentamycin solution at room temperature for 30 min. After being washed with PBS, PPs were weighed and homogenized. The diluted homogenate samples were plated on LB agar plates containing 25 μg ml^−1^ nalidixic acid (for *S*. Typhimurium* χ*3306) and 50 μg ml^−1^ kanamycin (for UF102 and Δ*FimH*), or on *Yersinia*-selective agar plates (Kanto Chemical Co.; for *Y. enterocolitica*). Data were excluded if no colonies were observed even in the highest amounts plating plate.

### BM transfer

BM cells were prepared from *Aif1*^*−/−*^mice (CD45.2) and congenic Ly5.1 mice (CD45.1). The cells were transferred to Ly5.1 and *Aif1*^*−/−*^mice, respectively, the haematopoietic cells of which had been disrupted by radiation (9.5 Gy). All mice were used for experiments 8 weeks after BM transfer. To confirm chimerism, flow cytometric analysis was performed to splenocytes prepared from all mice used in the experiments with antibodies to CD45.1 and CD45.2 (clones A40 and 104, respectively; BioLegend). At least 95% of the haematopoietic cells were replaced by donor cells (data not shown).

### Flow cytometric analysis of IgA-coated bacteria in faeces

Faeces were collected and weighed, and then suspended in PBS to 100 mg ml^−1^ by vigorous vortexing at 4 °C for 30 min. Each 500-μl suspension was centrifuged at 4 °C, 13,000 r.p.m. for 10 min. The pellet was then suspended in 1 ml 4% paraformaldehyde and incubated at 4 °C overnight with rotation. Each suspension was centrifuged at 4 °C, 1,000 r.p.m. for 2 min, and then 5 μl of the supernatant was washed twice with FACS buffer (2% FBS in PBS). Pellets were stained with FACS buffer containing 1 mg ml^−1^ rat serum (Jackson ImmunoResearch) and 1 μg ml^−1^ PE-conjugated anti-mouse IgA (clone mA-6E1, eBioscience) at 4 °C for 20 min, and were then analysed on NovoCyte Flow Cytometer Systems (ACEA). IgA-positive gate was determined by using isotype control antibody (Rat IgG1 κ, clone: RTK2071, BioLegend; Supplementary Fig. 7).

### Detection of *Y. enterocolitica-*specific antibody response

*Y. enterocolitica* (1 × 10^8^ CFUs) was given orally to BM-transferred chimeric mice once a week. Four weeks after the first inoculation, faeces were collected from the mice. Supernatants were prepared from 100 mg ml^−1^ diluted faeces, which were vigorously shaken and centrifuged at 4 °C. MaxiSorp plates (Thermo) were coated with 100 μl of 1.0 mg ml^−1^ lyophilized *Y. enterocolitica*. The plates were washed four times with 0.05% Tween20 in PBS (PBS-T) and then blocked with 1% BSA in PBS-T for 2 h at room temperature; the diluted samples were incubated for 2 h at room temperature. After the samples had been washed with PBS-T, they were incubated with HRP-conjugated anti-mouse IgA antibodies (Southern Biotech) for 2 h at room temperature. After washing of the samples with PBS-T again, a 3,3′,5,5′-tetramethylbenzidine Microwell Peroxidase Substrate system (Kirkegaard & Perry Laboratories) was used for detection according to the manufacturer's protocol. Absorbance was read at a wavelength of 450 nm with a SmartSpec Plus Spectrophotometer and an iMark Microplate Reader (Bio-Rad).

### Statistical analysis

Results were compared by using an unpaired two-tailed Student's *t*-test. Statistical significance was established at *P*<0.05. All statistical analyses were conducted by using GraphPad Prism 6. No statistical methods were used to determine sample size. These experiments were not randomized and the investigators were not blinded to allocation during experiments and outcome assessment.

### Data availability

The data that support the findings of this study are available from the corresponding author upon request.

## Additional information

**How to cite this article:** Kishikawa, S. *et al*. Allograft inflammatory factor 1 is a regulator of transcytosis in M cells. *Nat. Commun.* **8,** 14509 doi: 10.1038/ncomms14509 (2017).

**Publisher's note:** Springer Nature remains neutral with regard to jurisdictional claims in published maps and institutional affiliations.

## Supplementary Material

Supplementary Information Supplementary Figures

## References

[b1] Abreu M. T., Fukata M. & Arditi M. TLR signaling in the gut in health and disease. J. Immunol. 174, 4453–4460 (2005). 15814663 10.4049/jimmunol.174.8.4453

[b2] Fagarasan S. & Honjo T. Regulation of IgA synthesis at mucosal surfaces. Curr. Opin. Immunol. 16, 277–283 (2004). 15134775 10.1016/j.coi.2004.03.005

[b3] Madara J. L., Nash S., Moore R. & Atisook K. Structure and function of the intestinal epithelial barrier in health and disease. Monogr. Pathol. 31, 306–324 (1990). 2406578

[b4] Lievin-Le Moal V. & Servin A. L. The front line of enteric host defense against unwelcome intrusion of harmful microorganisms: mucins, antimicrobial peptides, and microbiota. Clin. Microbiol. Rev. 19, 315–337 (2006). 16614252 10.1128/CMR.19.2.315-337.2006 PMC1471992

[b5] MacDonald T. T. The mucosal immune system. Parasite Immunol. 25, 235–246 (2003). 12969442 10.1046/j.1365-3024.2003.00632.x

[b6] Neutra M. R., Mantis N. J. & Kraehenbuhl J. P. Collaboration of epithelial cells with organized mucosal lymphoid tissues. Nat. Immunol. 2, 1004–1009 (2001). 11685223 10.1038/ni1101-1004

[b7] Owen R. L. Uptake and transport of intestinal macromolecules and microorganisms by M cells in Peyer's patches--a personal and historical perspective. Semin. Immunol. 11, 157–163 (1999). 10381861 10.1006/smim.1999.0171

[b8] Vetvicka V., Tlaskalova-Hogenova H., Fornusek L., Rihova B. & Holan V. Membrane and functional characterization of lymphoid and macrophage populations of Peyer's patches from adult and aged mice. Immunology 62, 39–43 (1987). 3477526 PMC1453712

[b9] Reynolds J. D. Peyer's patches and the early development of B lymphocytes. Curr. Top. Microbiol. Immunol. 135, 43–56 (1987). 3495413 10.1007/978-3-642-71851-9_3

[b10] Iwasaki A. & Kelsall B. L. Freshly isolated Peyer's patch, but not spleen, dendritic cells produce interleukin 10 and induce the differentiation of T helper type 2 cells. J. Exp. Med. 190, 229–239 (1999). 10432286 10.1084/jem.190.2.229 PMC2195574

[b11] Kelsall B. L. & Strober W. Distinct populations of dendritic cells are present in the subepithelial dome and T cell regions of the murine Peyer's patch. J. Exp. Med. 183, 237–247 (1996). 8551227 10.1084/jem.183.1.237 PMC2192411

[b12] Kraehenbuhl J. P. & Neutra M. R. Epithelial M cells: differentiation and function. Annu. Rev. Cell Dev. Biol. 16, 301–332 (2000). 11031239 10.1146/annurev.cellbio.16.1.301

[b13] Terahara K. . Comprehensive gene expression profiling of Peyer's patch M cells, villous M-like cells, and intestinal epithelial cells. J. Immunol. 180, 7840–7846 (2008). 18523247 10.4049/jimmunol.180.12.7840

[b14] Hase K. . Uptake through glycoprotein 2 of FimH(+) bacteria by M cells initiates mucosal immune response. Nature 462, 226–230 (2009). 19907495 10.1038/nature08529

[b15] Nakato G. . Cutting Edge: brucella abortus exploits a cellular prion protein on intestinal M cells as an invasive receptor. J. Immunol. 189, 1540–1544 (2012). 22772447 10.4049/jimmunol.1103332

[b16] Sato S. . Transcription factor Spi-B-dependent and -independent pathways for the development of Peyer's patch M cells. Mucosal Immunol. 6, 838–846 (2013). 23212199 10.1038/mi.2012.122

[b17] Kanaya T. . The Ets transcription factor Spi-B is essential for the differentiation of intestinal microfold cells. Nat. Immunol. 13, 729–736 (2012). 22706340 10.1038/ni.2352 PMC3704196

[b18] de Lau W. . Peyer's patch M cells derived from Lgr5(+) stem cells require SpiB and are induced by RankL in cultured ‘miniguts'. Mol. Cell Biol. 32, 3639–3647 (2012). 22778137 10.1128/MCB.00434-12 PMC3430189

[b19] Utans U., Arceci R. J., Yamashita Y. & Russell M. E. Cloning and characterization of allograft inflammatory factor-1: a novel macrophage factor identified in rat cardiac allografts with chronic rejection. J. Clin. Invest. 95, 2954–2962 (1995). 7769138 10.1172/JCI118003 PMC295984

[b20] Mishima T. . Allograft inflammatory factor-1 augments macrophage phagocytotic activity and accelerates the progression of atherosclerosis in ApoE^−/−^ mice. Int. J. Mol. Med. 21, 181–187 (2008). 18204784

[b21] Ohsawa K., Imai Y., Kanazawa H., Sasaki Y. & Kohsaka S. Involvement of Iba1 in membrane ruffling and phagocytosis of macrophages/microglia. J. Cell Sci. 113, 3073–3084 (2000). 10934045 10.1242/jcs.113.17.3073

[b22] Ohsawa K., Imai Y., Sasaki Y. & Kohsaka S. Microglia/macrophage-specific protein Iba1 binds to fimbrin and enhances its actin-bundling activity. J. Neurochem. 88, 844–856 (2004). 14756805 10.1046/j.1471-4159.2003.02213.x

[b23] Autieri M. V., Kelemen S. E. & Wendt K. W. AIF-1 is an actin-polymerizing and Rac1-activating protein that promotes vascular smooth muscle cell migration. Circ. Res. 92, 1107–1114 (2003). 12714565 10.1161/01.RES.0000074000.03562.CC

[b24] Knoop K. A. . RANKL is necessary and sufficient to initiate development of antigen-sampling M cells in the intestinal epithelium. J. Immunol. 183, 5738–5747 (2009). 19828638 10.4049/jimmunol.0901563 PMC2922944

[b25] Casas I. A. & Dobrogosz W. J. Validation of the probiotic concept: Lactobacillus reuteri Confers broad-spectrum protection against disease in humans and animals. Microbial Ecology in Health & Disease 12, 247–285 (2000).

[b26] Walter J. Ecological role of lactobacilli in the gastrointestinal tract: implications for fundamental and biomedical research. Appl. Environ. Microbiol. 74, 4985–4996 (2008). 18539818 10.1128/AEM.00753-08 PMC2519286

[b27] Dramsi S. & Cossart P. Intracellular pathogens and the actin cytoskeleton. Annu. Rev. Cell Dev. Biol. 14, 137–166 (1998). 9891781 10.1146/annurev.cellbio.14.1.137

[b28] Reis R. S. & Horn F. Enteropathogenic Escherichia coli, Samonella, Shigella and Yersinia: cellular aspects of host-bacteria interactions in enteric diseases. Gut Pathog. 2, 8 (2010). 20649986 10.1186/1757-4749-2-8 PMC2921366

[b29] Kanazawa H., Ohsawa K., Sasaki Y., Kohsaka S. & Imai Y. Macrophage/microglia-specific protein Iba1 enhances membrane ruffling and Rac activation via phospholipase C-gamma -dependent pathway. J. Biol. Chem. 277, 20026–20032 (2002). 11916959 10.1074/jbc.M109218200

[b30] Rios D. . Antigen sampling by intestinal M cells is the principal pathway initiating mucosal IgA production to commensal enteric bacteria. Mucosal Immunol. 9, 907–916 (2016). 26601902 10.1038/mi.2015.121 PMC4917673

[b31] Isberg R. R. & Leong J. M. Multiple beta 1 chain integrins are receptors for invasin, a protein that promotes bacterial penetration into mammalian cells. Cell 60, 861–871 (1990). 2311122 10.1016/0092-8674(90)90099-z

[b32] Grutzkau A., Hanski C., Hahn H. & Riecken E. O. Involvement of M cells in the bacterial invasion of Peyer's patches: a common mechanism shared by Yersinia enterocolitica and other enteroinvasive bacteria. Gut 31, 1011–1015 (1990). 2210445 10.1136/gut.31.9.1011 PMC1378659

[b33] Autenrieth I. B. & Firsching R. Penetration of M cells and destruction of Peyer's patches by Yersinia enterocolitica: an ultrastructural and histological study. J. Med. Microbiol. 44, 285–294 (1996). 8606357 10.1099/00222615-44-4-285

[b34] Clark M. A., Hirst B. H. & Jepson M. A. M-cell surface beta1 integrin expression and invasin-mediated targeting of Yersinia pseudotuberculosis to mouse Peyer's patch M cells. Infect. Immun. 66, 1237–1243 (1998). 9488419 10.1128/iai.66.3.1237-1243.1998 PMC108039

[b35] Imai Y., Ibata I., Ito D., Ohsawa K. & Kohsaka S. A novel gene iba1 in the major histocompatibility complex class III region encoding an EF hand protein expressed in a monocytic lineage. Biochem. Biophys. Res. Commun. 224, 855–862 (1996). 8713135 10.1006/bbrc.1996.1112

[b36] Casimiro I., Chinnasamy P. & Sibinga N. E. Genetic inactivation of the allograft inflammatory factor-1 locus. Genesis 51, 734–740 (2013). 23929822 10.1002/dvg.22424 PMC3808495

[b37] Tsubata Y. . Expression of allograft inflammatory factor-1 in kidneys: a novel molecular component of podocyte. Kidney Int. 70, 1948–1954 (2006). 17035944 10.1038/sj.ki.5001941

[b38] Masuda T. . IRF8 is a critical transcription factor for transforming microglia into a reactive phenotype. Cell Rep. 1, 334–340 (2012). 22832225 10.1016/j.celrep.2012.02.014 PMC4158926

[b39] Minten C., Terry R., Deffrasnes C., King N. J. & Campbell I. L. IFN regulatory factor 8 is a key constitutive determinant of the morphological and molecular properties of microglia in the CNS. PLoS ONE 7, e49851 (2012). 23166780 10.1371/journal.pone.0049851 PMC3498170

[b40] Sibinga N. E., Feinberg M. W., Yang H., Werner F. & Jain M. K. Macrophage-restricted and interferon gamma-inducible expression of the allograft inflammatory factor-1 gene requires Pu.1. J. Biol. Chem. 277, 16202–16210 (2002). 11861656 10.1074/jbc.M200935200

[b41] Kierdorf K. . Microglia emerge from erythromyeloid precursors via Pu.1- and Irf8-dependent pathways. Nat. Neurosci. 16, 273–280 (2013). 23334579 10.1038/nn.3318

[b42] Schluesener H. J., Seid K., Kretzschmar J. & Meyermann R. Allograft-inflammatory factor-1 in rat experimental autoimmune encephalomyelitis, neuritis, and uveitis: expression by activated macrophages and microglial cells. Glia 24, 244–251 (1998). 9728770 10.1002/(sici)1098-1136(199810)24:2<244::aid-glia9>3.0.co;2-3

[b43] Pawlik A. . Association of allograft inflammatory factor-1 gene polymorphism with rheumatoid arthritis. Tissue Antigens 72, 171–175 (2008). 18721278 10.1111/j.1399-0039.2008.01086.x

[b44] Rochereau N. . Dectin-1 is essential for reverse transcytosis of glycosylated SIgA-antigen complexes by intestinal M cells. PLoS Biol. 11, e1001658 (2013). 24068891 10.1371/journal.pbio.1001658 PMC3775721

[b45] Niess J. H. . CX3CR1-mediated dendritic cell access to the intestinal lumen and bacterial clearance. Science 307, 254–258 (2005). 15653504 10.1126/science.1102901

[b46] Vallon-Eberhard A., Landsman L., Yogev N., Verrier B. & Jung S. Transepithelial pathogen uptake into the small intestinal lamina propria. J. Immunol. 176, 2465–2469 (2006). 16456006 10.4049/jimmunol.176.4.2465

[b47] Ley K., Laudanna C., Cybulsky M. I. & Nourshargh S. Getting to the site of inflammation: the leukocyte adhesion cascade updated. Nat. Rev. Immunol. 7, 678–689 (2007). 17717539 10.1038/nri2156

[b48] Tian Y. & Autieri M. V. Cytokine expression and AIF-1-mediated activation of Rac2 in vascular smooth muscle cells: a role for Rac2 in VSMC activation. Am. J. Physiol. Cell Physiol. 292, C841–C849 (2007). 16987989 10.1152/ajpcell.00334.2006

[b49] Gulig P. A. & Curtiss R. 3rd Plasmid-associated virulence of Salmonella typhimurium. Infect. Immun. 55, 2891–2901 (1987). 3316027 10.1128/iai.55.12.2891-2901.1987 PMC260003

[b50] Gulig P. A., Doyle T. J., Hughes J. A. & Matsui H. Analysis of host cells associated with the Spv-mediated increased intracellular growth rate of Salmonella typhimurium in mice. Infect. Immun. 66, 2471–2485 (1998). 9596705 10.1128/iai.66.6.2471-2485.1998 PMC108227

[b51] Datsenko K. A. & Wanner B. L. One-step inactivation of chromosomal genes in Escherichia coli K-12 using PCR products. Proc. Natl Acad. Sci. USA 97, 6640–6645 (2000). 10829079 10.1073/pnas.120163297 PMC18686

