# Supplementary material for: Allograft inflammatory factor 1 is a regulator of transcytosis in M cells
Source: Nat Commun. 2017 Feb 22;8:14509. doi: 10.1038/ncomms14509 (PMC5322540; doi:10.1038/ncomms14509)
Supplement: Supplementary Information — Supplementary Figures [file ncomms14509-s1.pdf]

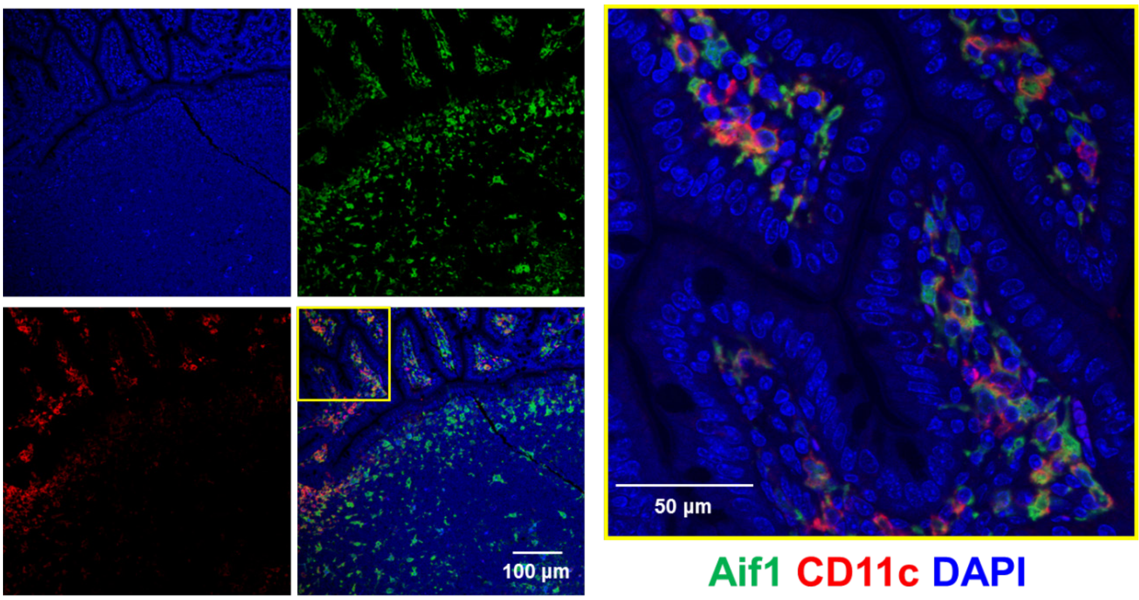

Supplementary Figure 1. Aif1 expression in CD11c-positive cells.
Immunofluorescence staining of PPs prepared from a C57BL/6J mouse with anti-Aif1 antibody (green) and anti-CD11c antibody (red), and counterstaining with 4′,6-diamidino-2-phenylindole (DAPI) (blue). Data are from one experiment representative of three independent experiments.

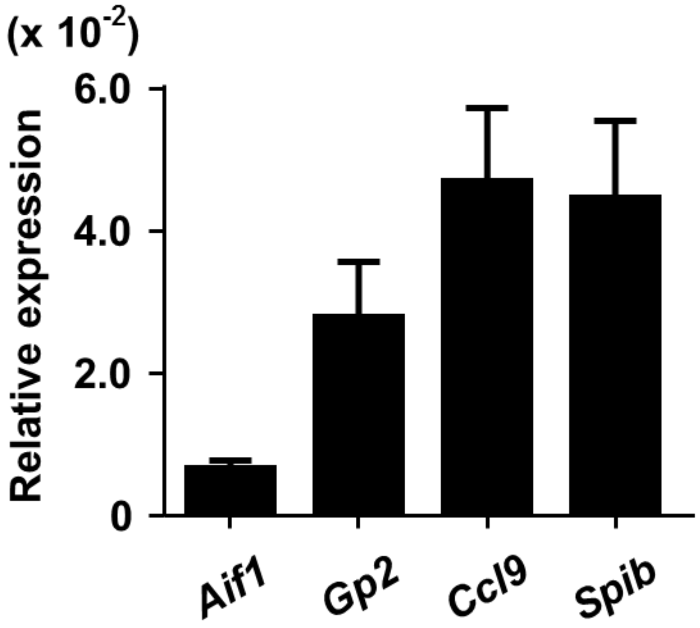

Supplementary Figure 2. Comparison of expression levels of *Aif1* and M-cell index genes.

Total RNA samples were prepared from FAE of C57BL/6J mice. Relative expression of the indicated genes was determined by quantitative PCR. Each result was normalized against the expression of *Gapdh.* Data are shown as means±SEM (triplicate) from one experiment representative of two independent experiments.

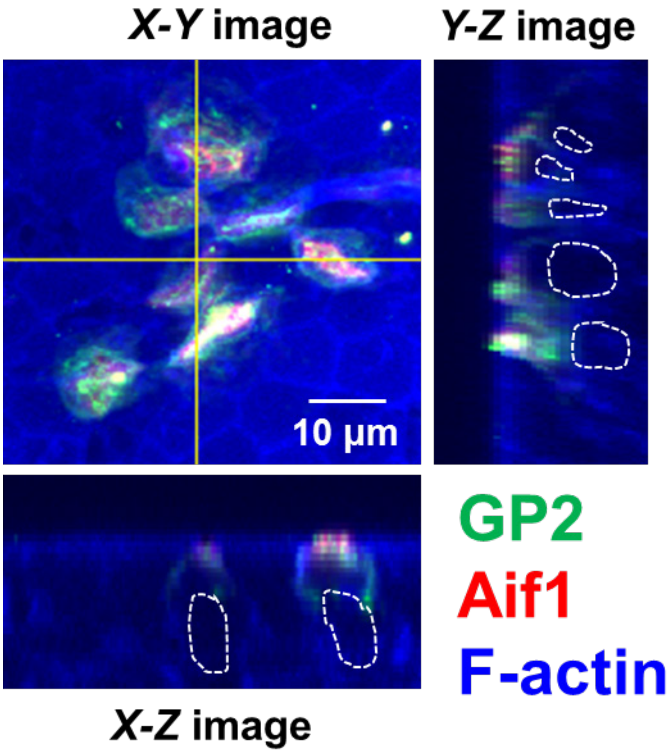

Supplementary Figure 3. Localization of Aif1 in M cells.

PPs prepared from C57BL/6J mouse were whole-mount stained for GP2 (green), Aif1 (red) and F-actin (blue). Serial *X-Y* images of whole-mount staining were obtained with confocal laser microscope, and then *X-Z* and *Y-Z* images were reconstituted as a transverse image. Dotted circles indicate a pocket structure of M cells. Data is from one experiment representative of two independent experiments.

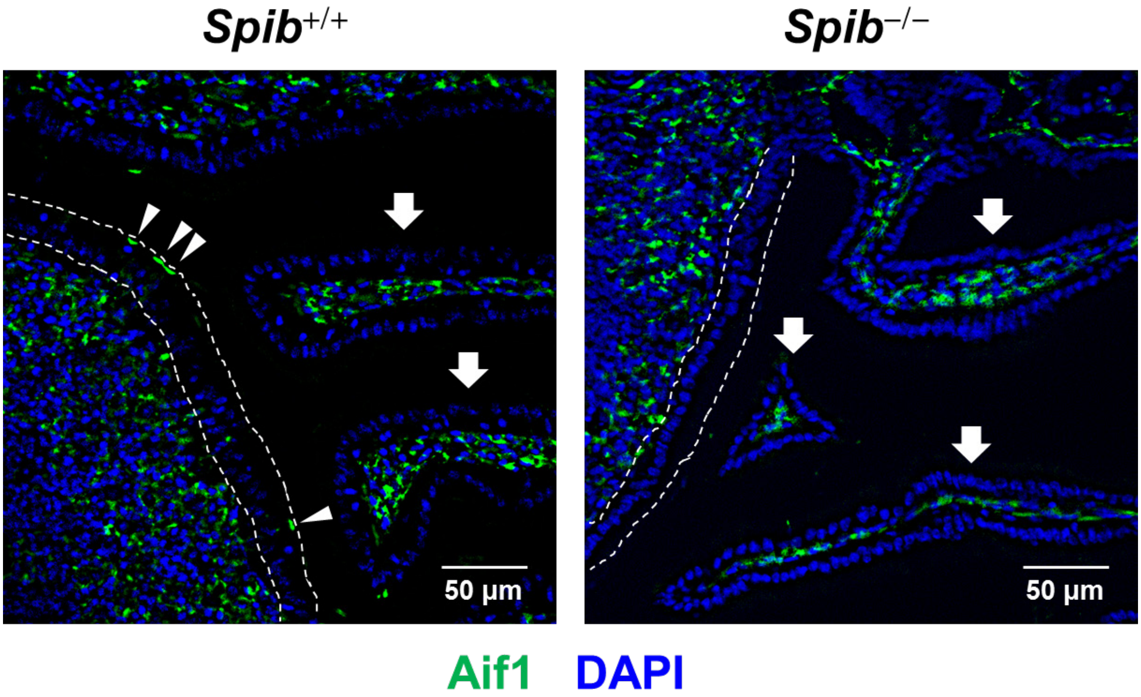

Supplementary Figure 4. Aif1 expression in *Spib*$^{-/-}$ PPs and lamina propria. Immunofluorescence staining of PP and villi prepared from *Spib*$^{+/+}$ and *Spib*$^{-/-}$ mice with anti-Aif1 antibody (green), and counterstaining with DAPI (blue). Data are from one experiment representative of three independent experiments. Dotted lines, arrowheads, and arrows indicate FAE regions, Aif1-positive M cells, and villi (lamina propria), respectively.

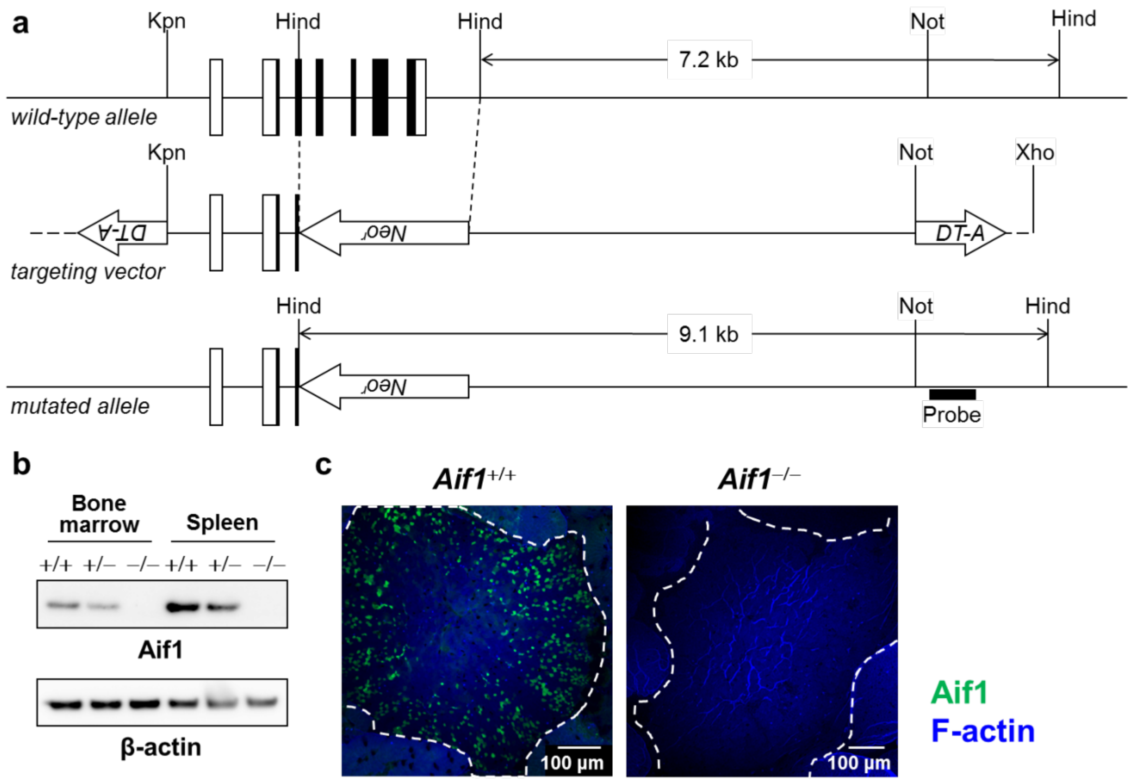

Supplementary Figure 5. Targeting strategy of the mouse *Aif1* gene.

(**a**) Schematic representation of the targeting strategy. Open and filled boxes indicate *Aif1* noncoding and coding exons, respectively. A targeting construct was designed to replace part of exon 3 to exon 7 of *Aif1* to neomycin resistance gene cassette (*Neo*^{r}). The closed bar indicates the probe for Southern screening of ES cells. Relevant Kpn I (Kpn), Hind III (Hind), Not I (Not), and Xho I (Xho) recognition sites are indicated. (**b**) Western blot analysis of Aif1 protein expressed in primary bone-marrow or splenic cells prepared from mice of the indicated *Aif1* genotypes. Cells were lysed, subjected to SDS-PAGE and immunoblotted with rabbit anti-mouse Aif1 antibody. The same membrane was re-probed with anti-β-actin antibody in the case of the internal controls. Data are from one experiment representative of two independent experiments. (**c**) Whole-mount staining for Aif1 (green) in the FAE of *Aif1*$^{+/+}$ and *Aif1*$^{-/-}$ mice. Dotted lines indicate the peripheral of FAE region. Data are from one experiment representative of three independent experiments.

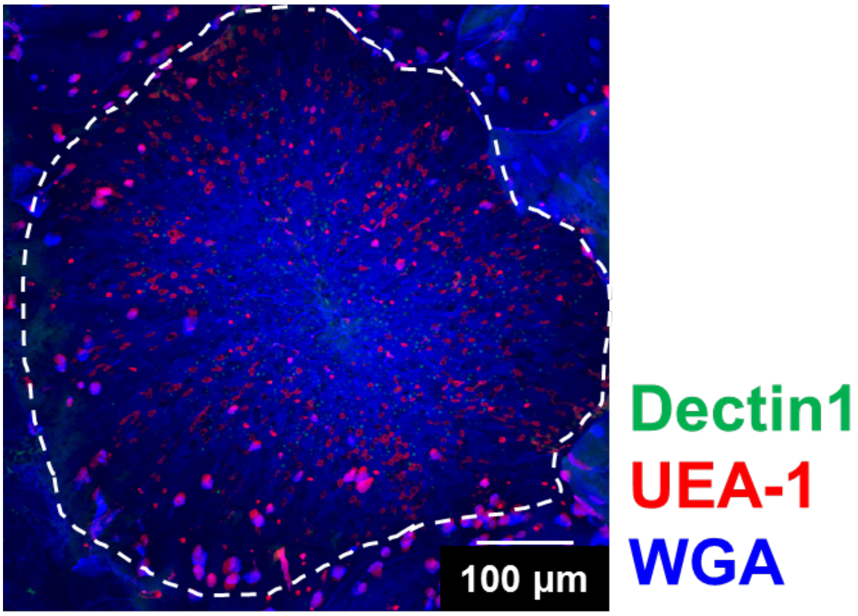

Supplementary Figure 6. Dectin-1 is not expressed in FAE.

Whole-mount Dectin-1 staining image of C57BL/6J PPs is shown. UEA-1 and WGA lectins were used for counterstaining. Dotted lines indicate the peripheral of FAE region. Data are from one experiment representative of two independent experiments.

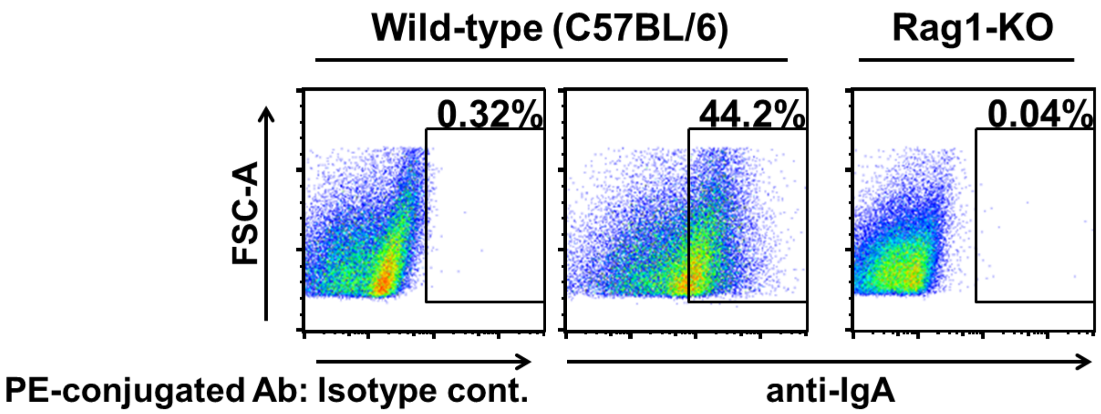

Supplementary Figure 7. Determination of flow cytometry gate to detect IgA-coated bacteria.

We determined ‘IgA-positive’ gate by using faecal samples prepared from wild-type mice and isotype control antibody (left panel). IgA-coated bacteria were actually detected in this gate in faecal samples prepared from wild-type mice (middle panel), but not in the samples prepared from Rag1-deficient mice (right panel). This gate setting was always done before the experiments.

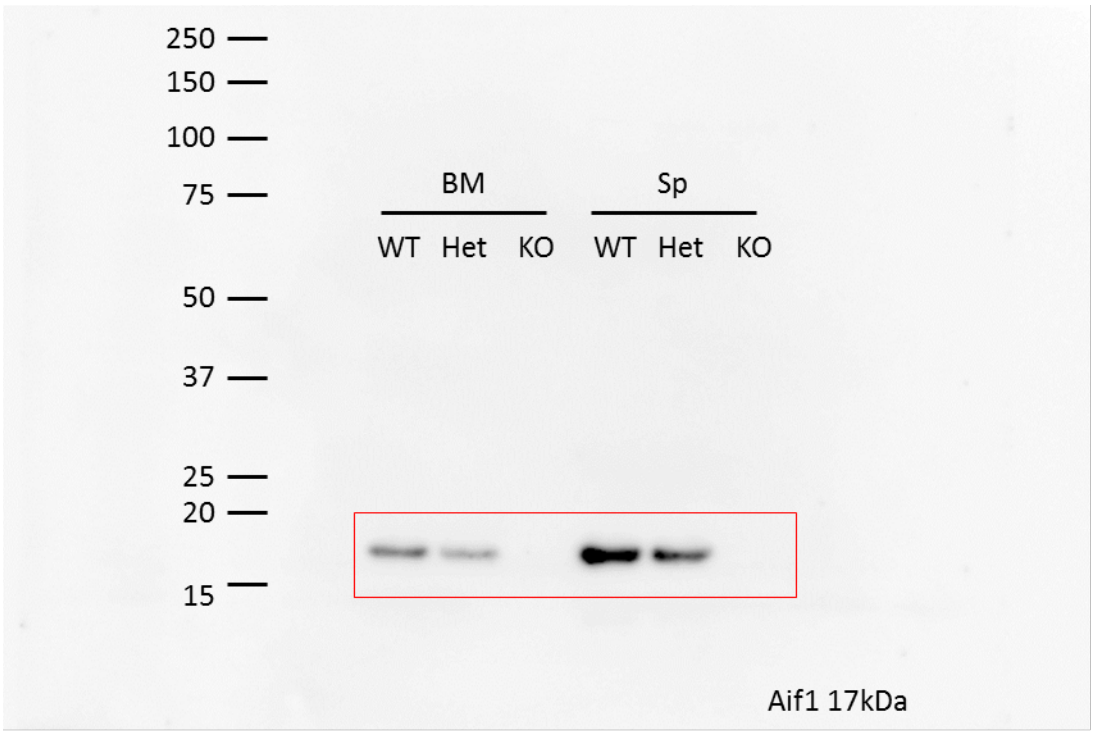

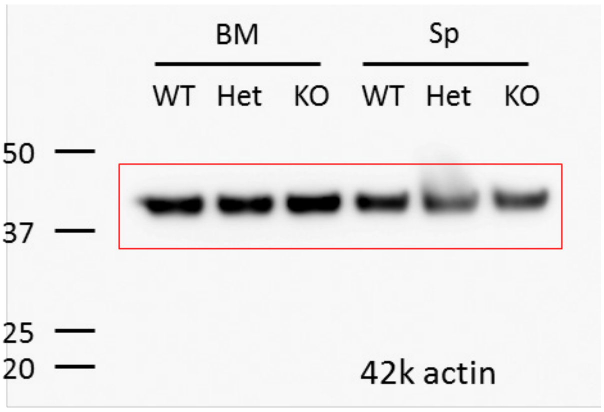

Supplementary Figure 8. Full-size images of Western blots.

Red square regions were used as Supplementary Figure 5b.
